# Gender differences in understanding and acceptance of robot-assisted surgery

**DOI:** 10.1007/s11701-019-00960-z

**Published:** 2019-05-02

**Authors:** Hilary McDermott, Nazmin Choudhury, Molly Lewin-Runacres, Ismail Aemn, Esther Moss

**Affiliations:** 1 School of Sport, Exercise and Health Sciences, Loughborough University, Loughborough, Leicestershire LE11 3TU UK; 2 Department of Gynaecological Oncology, University Hospitals of Leicester, Leicester, Leicestershire UK; 3 Leicester Cancer Research Centre, University of Leicester, Leicester, LE2 7LX UK

**Keywords:** Robot-assisted surgery, Gender, Acceptance

## Abstract

Robot-assisted surgery has numerous patient benefits compared to open surgery including smaller incisions, lower risk of infection, less post-operative pain, shorter hospital stays and a quicker return to the workforce. As such, it has become the first-choice surgical modality for several surgical procedures with the most common being prostatectomy and hysterectomy. However, research has identified that the perceptions of robot-assisted surgery among surgical patients and medical staff often do not accurately reflect the real-world situation. This study aimed to understand male and female perceptions of robot-assisted surgery with the objective of identifying the factors that might inhibit or facilitate the acceptance of robotic surgery. Semi-structured interviews were undertaken with 25 men/women from diverse social/ethnic backgrounds. The interviews were transcribed and analysed using thematic analysis. The majority of female participants expressed concerns in relation to the safety and perception of new technology in surgery, whereas many male participants appeared to be unfazed by the notion of robotic surgery. There were clear differences in how males and females understood and conceptualised the robot-assisted surgical process. Whilst male participants tended to humanise the process, female participants saw it as de-humanising. There is still a discrepancy between the public perceptions of robotic surgery and the clinical reality perceived by healthcare professionals. The findings will educate medical staff and support the development of current informative techniques given to patients prior to surgery.

## Introduction

Advances in technology have been increasingly adopted in surgery. The introduction of laparoscopic surgery marked the birth of minimally invasive surgery (MIS) and placed a technology interface between surgeons and their patients [22]. MIS has numerous patient benefits as compared to open surgery, including smaller incisions, lower risk of infection and less post-operative pain, shorter hospital stays, a quicker return to the workforce and has become the first-choice modality for numerous procedures across many surgical specialties [4, 11, 16].

Robot-assisted surgery (RS) involves a surgeon at a console operating remote-controlled robotic arms which facilitates the performance of laparoscopic procedures [5]. RS was first undertaken in general surgery in 2001 [26] and presents an alternative minimally invasive modality [25]. Substantial surgical advantages are reported with this method including improved dexterity, reduction/elimination of tremors, as well as superior visualisation including three-dimensional imaging [13, 15, 18]. Studies have also identified that RS has the potential to shorten the learning curve as compared to straight-stick laparoscopy for trainee surgeons [8].

Currently the most common RS procedures are prostatectomy and hysterectomy. However, research has consistently demonstrated that patient understanding of robotic-assisted surgery is poor. Irani et al. [14] examined patient knowledge and understanding of the differences between open, laparoscopic and robotic-assisted surgery and found that 34% of participants did not understand the difference between open and laparoscopic surgeries and 46% of the participants did not understand the difference between laparoscopic and robotic procedures. Furthermore, 67.5% of participants were not aware that the surgeon directly controls the movement of one or more robotic arms. The authors acknowledged that differences in education and surgical history may account for some of these differences, but not all of them, and concluded that health care providers should not assume that patients have an adequate understanding of their surgical options and should educate them about those options in order that they can make informed decisions [14]. Ahn et al. [2] surveyed 208 female gynaecological patients and identified gaps and inconsistencies in patient knowledge surrounding modes of surgical interventions. They concluded that such gaps can lead to misconceptions.

Research has also identified that the perceptions of robotic surgery among surgical patients and medical staff often do not accurately reflect the real-world situation [1] and there is widespread misunderstanding of the role of the robot [7]. Boys et al. [7] reported that 67% of their participants thought the robot could malfunction during surgery thus causing internal damage to the patient, 15% thought the robot could perform the wrong operation and 55% reported that they would rather have the conventional minimally invasive surgery rather than having robotic surgery. Participants also displayed misperceptions about the role of the surgeon with 20% believing that the surgeon would stand and watch over whilst the robot carried out the procedure with its autonomous function. Boys’s study was one of the first to focus on the perceptions of robot-assisted surgery among the general public and utilised a web-based survey. However, 94% of respondents were from the United States and over half (53%) had a background in health care and 13% were physicians [7]. It is, therefore, apparent that further studies are needed to fully understand public interest and perception in relation with robot-assisted surgery.

Shared decision making in healthcare services involves the collaboration of patients and clinicians in selecting tests and treatment options based on the clinician’s expertise and the informed preferences of the patients [3]. This is important especially in the modern UK healthcare system, where shared decision making is an essential component of patient care. This collaboration requires healthcare providers to work alongside patients to ensure that the healthcare system remains safe and patient-centred [20] and includes understanding a patient’s emotional needs to allay any fear and anxiety [10]. Knowledge of specific patient preferences is needed, so that care can be modified to meet the needs of the patient [26]. This is particularly important in robotic-assisted surgery, where despite the well-documented clinical benefits of this procedure, there are obstacles to patient acceptance, since new technology can often generate fear and apprehension amongst patients, because they perceive technology differently to surgeons and clinicians [23].

Given this fear and the reported widespread misunderstanding about robot-assisted surgery, this study utilised semi-structured interviews to gain insight into male and female perceptions of robot-assisted surgery with the objective of identifying the factors that might inhibit or facilitate the acceptance of robotic surgery among participants.

## Methods

Ethical approval was given for this study by Loughborough University Ethical Advisory Committee. An interview schedule was developed at the beginning of the study, which was informed through engagement with the literature, and was piloted before producing the final version. Open-ended questions were utilised in conjunction with probing questions to elicit deep, meaningful data to encourage further discussion and to clarify participant responses. The interview schedule focused on gynaecological surgery for female participants and prostrate surgery for male participants.

### Sampling

Sampling was on a convenience basis and was used to recruit participants from diverse, backgrounds to capture their understandings of the research area. Participant selection was made on the basis that they were over the age of 18 and from a variety of ethnic backgrounds. An online advertisement was posted on social media (Facebook) outlining details of the study. Snow-ball sampling was also utilised as volunteers can sometimes skew data as they share similar characteristics [19].

### Procedure

The interviews were undertaken by two researchers (NC and ML) both of whom had been trained in interview techniques. Prior to the interviews, participants were presented with a participant information sheet and informed consent was obtained from all individual participants. The interviews were audio recorded with the knowledge and consent of the interviewee and lasted between 20 and 30 min.

### Analysis

The recorded interviews were transcribed and coded accordingly following the phases of thematic analysis outlined by Braun and Clarke [6]. This approach was used due to accessibility and theoretical flexibility when interpreting patterns and themes [6]. The first phase involved immersion in the data by reading and rereading the transcripts to the extent that the researcher is familiar with the breadth and depth of the dataset before establishing patterns. These patterns are then systematically labelled with meaningful codes, and related codes were collated together. The third phase involved synthesising and organising relevant codes into themes and sub-themes that are meaningful. This was achieved by creating thematic maps, where codes were arranged into a number of themes that capture important aspects related to the research question. Throughout the analysis, the codes were continually reviewed and refined into key themes/sub-themes and altered following further interpretation.

## Results

In total, 25 face-to-face semi-structured interviews were undertaken with 12 males and 13 females (Table 1).

**Table 1:** Participant demographics

Demographics	Number (percentage)
Age (mean)	21.5 (19–26)
Sex	
Female	13
Male	12
Ethnicity	
White British	16
British–Bangladeshi	3
British African	2
British Indian	3
British Pakistani	1

### Acceptance of new technology

The majority of female participants expressed concerns in relation with the safety and perception of new technology in surgery, whereas many male participants appeared to be unfazed by the notion of robotic surgery. Most male participants had heard of robotic-assisted surgery, yet only two demonstrated a correct awareness of the role of the surgeon. One 19-year-old male who had not heard of robotic-assisted surgery prior to the interview seemed to accept the concept easily due to the ubiquitous nature of technology:

‘[Robotic surgery] is just one of those things that I kind of assumed was real […] you can get robotic everything now’.

Although the majority of males had heard of robotic-assisted surgery, there was a clear lack of understanding as to the surgeon’s role in RS with one 20-year-old male stating:

‘It almost calls into question what the point is, if you’ve got a fully qualified surgeon in the room then why leave it up to the robot?’

The lack of acceptance of new technology expressed by most female participants appeared to be based on trust. A 26-year-old female stated:

‘First of all, it’s the first time I’ve heard that, and as humans, we tend to trust what’s been there traditionally and erm it’s obviously a new method and I am quite sceptical about modern technology and so I would not trust a robot”

Despite the male participants being more accepting of new technology, trust was also an evident important concept. One 20-year-old male stated:

‘I trust humans more than I trust robots in matters like this’

A further 19-year-old male added:

‘I worry about the machine just failing, anything could happen […] some people have the idea that robots can create a mind of their own, so what if the robot takes over?’

The media were described as being an important source of information for both male and female participants in relation to attitudes towards the acceptance of new technology in medicine. For some participants, the media were an effective tool in increasing awareness and understanding and acceptance, but for others, the media resulted in misconceptions association with the term ‘robot’. For example, one 22-year-old female participant characterised robotic surgery as:

‘Metal? With a weird face and long arms, I don’t know what it looks like. Erm and then it might have sharp thing to cut you open I just thinking of horror movies’

A further 23-year-old female participant stated:

‘I just think about robots from movies. In the media robots are perceived as not having human intelligence so I think they are doing as they are told. But I would imagine it as hands, feet and eyes’

Some male participants also associated the concept of RS with the traditional concept of robots. A 23-year-old male stated:

‘When I think of robots I think of Daleks and big clunky things’

This suggests that an important factor associated with trust may be a fear of malfunction. This also suggests that there is a lack of knowledge in understanding the role of a surgeon when performing RS. A 24-year-old female described her understanding:

‘I can assume that it would be a surgery that is being taking place via a robot. So something that is automatic that does not require human interaction like a surgeon taking the operation. […] I am just imagining like we use […] artificial intelligence’

A further female, aged 23 stated:

‘I just think about robots from movies. In the media robots are perceived as not having human intelligence so I think they are doing as they are told. But I would imagine it as hands, feet and eyes’

### (De)humanisation of robotic surgery

When discussing RS, there were clear differences in how males and females understood and conceptualised the surgical process. Whilst male participants tended to humanise the process, female participants saw it as de-humanising. This was largely, because modern technology can dehumanise the surgeon’s role in the patient care, thus creating a perception of clinical distance and dissociation between the doctor and patient.

Many female participants reported that the doctor–patient relationship was considered an essential aspect of patient care prior to undergoing an operation. Being able to communicate and interact socially with the surgeon was associated with reassurance and building trust. These participants argued that a robot was unable to meet these criteria, as it lacked the qualities of a human surgeon, for example, a 24-year-old female stated:

‘I personally like social interaction. […] I would like to know the surgeon that is doing my operation […] there is something satisfying in meeting someone face to face. That whole element of human interaction or socialising […] is not there with robots. How am I supposed to get that satisfaction from a robot? I am going to be getting it from a third person that will tell me what the robot will be doing.’

Males on the other hand humanised surgical robots and exhibited a sense of anthropomorphism in relation with robotic surgery. For example, a 19-year-old male stated:

‘A human only has two hands and can only do one thing at once but those guys can do five different things at the same time’.

It is interesting that this male participant used masculine terms when referring to the robot itself. Interviewees who used language likening robots to humans were more likely to be accepting and less frightened of the concept of robotic surgery. It is plausible that imagining the robot as a human helps to eliminate fear and compensate for the perceived clinical distance. This was demonstrated by one participant, a 20-year-old male, whom when asked to explain what he imagined when hearing the word ‘robot’:

‘I see a sort of man but in robot form’

This participant combined the imagery of a human and a machine to create his perception of the actual robot used in surgery, making the surgical process more humanised.

## Discussion

The aim of this study was to explore men and women’s perception and attitudes towards RS. This research has identified that there is a general lack of understanding of RS among young lay men and women. Such misunderstanding can inhibit the acceptance of this surgical approach and invoke a fear of technology typically derived from traditional concepts of ‘robots’. General fear and misconception towards robotic procedures were found to be internalised by stereotypical and conventional representations from the media, as participants were left to rely on myth. Those participants with a greater level of knowledge and awareness were more likely to be more accepting of robotic-assisted surgery.

In line with the previous research [1], participants displayed negative attitudes and perception towards the concept robotic-assisted surgery. Such perceptions were associated with the robot being perceived as an artificial intelligence with automated functions to carry out invasive procedures, without the assistance of a surgeon. This supports work by Irani et al. [14], where more than 65% of the participants were unaware that the surgeon directly controls the manoeuvre of one or more robotic arms through a computer-controlled system.

Participants also expressed deep-rooted concerns over the acceptance of new technology, as it would create a clinical distance and dissociation between the surgeon and patient. This was reported to reduce personal interaction, as the robot was unable to meet the needs of specific patient preferences, thereby reducing patients’ trust [26].

The findings of this study can be underpinned by the Unified Theory of Acceptance and Use of Technology model (UTAUT) [24], as the four-core constructs of performance expectancy, effort expectancy, social influence, and facilitating conditions were all discussed by participants. Performance expectancy was highlighted in responses which suggested a fear of malfunction or a lack of trust towards the capabilities of robotics. The majority of participants did not demonstrate a full awareness of robot-assisted surgery and as such may have perceived effort expectancy to be high as significant time would be required to research the concept. Social influences, in particular media representations, were apparent in driving participant’s conceptualisation and personal attitudes were evident in the results.

This study is, to the authors’ knowledge, the first to investigate lay perceptions of robot-assisted surgery in relation with UTAUT. The findings suggest an overall fear and lack of trust of new technology in medicine and this was greater among lay women than men. This is an important finding for medicine, as this may prevent patients from accepting robot-assisted surgery over other methods. It is also interesting that these views were expressed given the age of the participants, where the incorporation of technology into everyday life appears to be ubiquitous.

Despite the potential limitations, the findings in this study demonstrate support for existing research on patient’s perception towards robotic surgery [1, 7]. Moreover, the use semi-structured interviews enabled the researcher to capture meaningful data and probe further discussion on sensitive topics, which may not have been achieved if questionnaires or surveys were utilised as primary data collection [12]. Shauver and Chung [21] argue that semi-structured interviews are suited to investigating complex and sensitive issues, such as patient satisfaction or how patients and family make treatment decisions. Convenience sampling was employed due to the selection method being cost minimal, and to facilitate easy access to participants [17]. In addition, the use of convenience sample is suited for exploratory purposes, as it enables the researcher to get different views of a problem and explore constructs for a particular issue [9]. According to Ferber [9], data obtained from a convenience sample can convey feelings of realism than other sampling methods such as random or voluntary. A variety of methods were adopted to recruit participants to achieve a structured convenience sample. The mean age of participants was 21.5 years, and whilst this study reflects the attitudes of a young population, the findings are insightful and offer a useful lens to examine patient understanding of robot-assisted surgery.

## Conclusions

The findings from this study identify that there is still a discrepancy between the public perceptions of robot-assisted surgery and the clinical reality perceived by healthcare professionals. The findings will educate medical staff and support the development of current informative techniques given to patients prior to surgery.
